# Acute kidney injury decreases long-term survival over a 10-year observation period

**DOI:** 10.1186/cc12903

**Published:** 2013-11-05

**Authors:** Adam Linder, Adeera Levin, Keith Walley, James A Russell, John H Boyd

**Affiliations:** 1 Centre for Heart Lung Innovation, Division of Critical Care Medicine, St Paul's Hospital, University of British Columbia, Vancouver, BC, Canada; 2 Division of Nephrology, St Paul's Hospital, University of British Columbia, Vancouver, BC, Canada

## Background

We hypothesized that one single episode of acute kidney injury (AKI) reduces long-term survival compared with no acute kidney injury (No AKI) following recovery from critical illness.

## Materials and methods

A prospective cohort of 2,010 patients admitted to the ICU between 2000 and 2009 at a provincial referral hospital was followed to determine whether AKI influences long-term survival.

## Results

**Figure 1:**
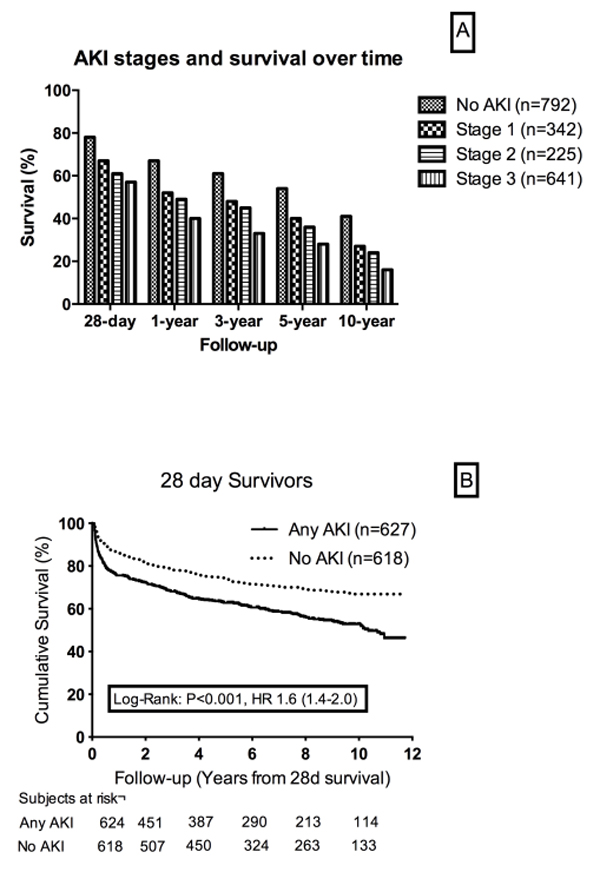
***A: *Bar chart showing that patients with AKI of any stage had significantly poorer mean survival rates compared to control patients with no AKI, at 28-days, 90-days, 1-year, 3-years, 5-years and 10-years after enrolment**. *B: *Unadjusted Kaplan-Meier curves showing the10-year survival from ICU admission for patients classified as having any stage of AKI according to the KDIGO classification using serum creatinine. Time is calculated from 28 days after admission (28-day survivors). Mantel-Cox Log Rank showed a significant difference in mortality between the two curves with or without AKI.

Of the 1,844 eligible patients, 18.4% had AKI stage 1, 12.1% had stage 2, 26.5% had stage 3, and 43.0% had No AKI, using the KDIGO classification. The mean and median follow-up time was 8.1 and 8.7 years. The 28-day, 1-year, 5-year and 10-year survival rates were 59.6%, 44.9%, 37.4%, and 33.4%, in patients with any AKI (stage 1, stage 2, stage 3), which was significantly worse compared with the critically ill patients with no AKI at any time (*P *< 0.01). The adjusted 10-year mortality risk associated with AKI was 1.44 (95% CI = 1.2 to 1.7) among 28-day survivors. Patients who had mild AKI (stage 1) had significantly worse survival at 28 days, 1 year, 3 years, 5 years and 10 years compared with No AKI (*P *< 0.01) (Figure 1A). Patients with sepsis and AKI who survived 28 days had significantly poorer 5-year and 10-year survival compared with nonseptic AKI (*P *< 0.01) (Figure 1B).

## Conclusions

Patients with one episode of mild (stage 1) AKI have significantly lower survival rates over 10 years than critically ill patients without AKI. The causes and mechanisms of this association warrant further careful study. Close medical follow-up of these patients may be warranted and mechanistic research required understanding how AKI influences distant events.

